# Impact of vaccines on antimicrobial resistance

**DOI:** 10.1099/jmm.0.002050

**Published:** 2025-08-14

**Authors:** Gordon Dougan, Emily Hugo-Webb

**Affiliations:** 1 Microbiology Society, London, UK

**Keywords:** antimicrobial resistance, One Health, public health, vaccines

## Abstract

Antimicrobial resistance (AMR) is a real and current threat to public health, yet the role of vaccines in combating this crisis remains underutilized and under-recognized. This meeting report summarizes key insights from a multidisciplinary workshop convened by the Microbiology Society in February 2025, as part of the Knocking Out AMR initiative, bringing together 21 expert stakeholders across academia, industry, clinical and veterinary sectors and policy. The workshop explored how vaccines can reduce the burden of AMR by preventing infections, limiting antibiotic use and slowing resistance development. Discussions highlighted the need to strengthen the evidence base for vaccine-mediated AMR reduction, address policy and regulatory barriers and incentivize public–private collaboration in vaccine development. Participants called for AMR impact to be formally recognized in vaccine labelling and national immunization strategies, and for greater integration of vaccines into AMR action plans. The workshop also underscored the importance of One Health approaches, investment in research for both human and animal vaccines and the role of the microbiology community in driving change.

## Introduction from the President

As President of the Microbiology Society and someone who has spent my career working at the intersection of infectious disease, microbiology and public health, I was honoured to chair this timely and important workshop on the role of vaccines in addressing antimicrobial resistance (AMR).

AMR is no longer a looming threat: it is a present-day crisis affecting lives, healthcare systems and economies around the world. Yet, despite the scale of the problem, we have not fully harnessed one of our most powerful tools: vaccines. Vaccines prevent infections before they occur, reducing the need for antibiotics and slowing the emergence of resistance. Their potential in tackling AMR is well known in principle but still poorly recognized in policy and practice.

This workshop was designed to change that. We brought together a diverse group of experts, spanning human and animal health, academia, industry and policy, to confront the barriers and chart a more coordinated path forward. From scene-setting talks to breakout sessions, it was clear that whilst challenges remain, the appetite for collaboration and action is strong.

This report captures the rich discussions and practical recommendations that emerged from the day. My hope is that it will serve as a foundation for future efforts, not only within the microbiology community, but across the wider public health landscape. The fight against AMR demands bold thinking and sustained commitment. Vaccines must be part of that conversation, and I’m proud that the Microbiology Society is helping to lead the way through the Knocking Out AMR project.

## Workshop overview

### Attendees

Experts in the field were invited to a full-day workshop on 13 February 2025 at the Microbiology Society office in London. The workshop was chaired by Professor Gordon Dougan (University of Cambridge) and President of the Microbiology Society.

The workshop was attended by 21 expert stakeholders from a range of sectors including academia, industry, clinical and veterinary settings, regulatory bodies, funding bodies and national and international policy agencies. Attendees also covered both human and animal health and a wide range of micro-organisms (bacteria, fungi, viruses and parasites).

### Structure

The workshop was structured into two main sessions:

- The morning session focused on setting the stage for discussions on the role of vaccines in mitigating AMR. It began with an overview of the event’s aims, followed by a series of scene-setting presentations from leading experts representing academia, funding bodies, veterinary science and both international and UK policy perspectives. These talks explored scientific, financial, regulatory and practical challenges related to AMR-focused vaccines. Throughout the session, speakers and attendees engaged in an in-depth conversation on key issues, including strengthening the evidence base for vaccines in AMR strategies, overcoming regulatory hurdles and fostering industry collaboration.
- The afternoon session featured breakout group discussions, where attendees explored how the microbiology community and the UK Government can take action to prioritize vaccines as a tool to tackle AMR. The event concluded with a feedback session and wrap-up, summarizing key takeaways and potential next steps.

## Workshop summary

### Scene-setting presentations

#### Academic perspective – Professor Miguel Valvano (Queen’s University Belfast)

Miguel Valvano provided an overview of the scientific basis for vaccine-driven AMR reduction. Key points included the following:

- How vaccines reduce antibiotic use by preventing bacterial infections.

- The need for more research into vaccines targeting priority AMR pathogens such as *Klebsiella pneumoniae* and *Escherichia coli*.
- Challenges in developing broad-spectrum vaccines that address multiple resistant strains.

#### Funding perspective – Professor Adam Cunningham (University of Birmingham, Co-Director of the BactiVac Network)

Adam Cunningham discussed the funding landscape for AMR-related vaccines. Key points included the following:

- Current funding gaps in vaccine development for AMR. Whilst funding for vaccine development is increasing, AMR-targeted vaccines often struggle to attract investment due to the long timelines and complex clinical trials.
- The role of networks like BactiVac in fostering industry-academic partnerships.
- The need for more investment in translational research to bridge preclinical and clinical vaccine development.

#### Veterinary perspective – Professor Fiona Tomley (The Royal Veterinary College)

Fiona Tomley provided insights on the role of vaccines in reducing AMR in animal health. Key points included the following:

- How livestock vaccination reduces antibiotic consumption in food production.
- The importance of One Health approaches in tackling AMR across human, animal and environmental sectors.
- The need for better vaccines against bacterial infections in poultry, cattle and pigs, which are major sources of antibiotic use.
- The need for more research on AMR-resistant bacterial strains in veterinary medicine.

#### International policy perspective – Dr Mateusz Hasso-Agopsowicz (World Health Organization)

Mateusz Hasso-Agopsowicz provided insights into the World Health Organization’s (WHO) efforts to integrate vaccines into global AMR strategies. Key points included the following:

- The impact of vaccines on AMR. Mateusz summarized the WHO’s report estimating the impact of vaccines on AMR [1], which highlights the significant potential benefits of vaccines: 515,000 deaths averted, $28 million saved, $30 billion in hospital cost reductions, $20 billion in productivity savings and 2.5 billion fewer antibiotic doses used.
- Despite their significant potential, vaccines are still under-utilized and under-recognized as a tool in the fight against AMR. Their role in reducing antibiotic use and preventing resistant infections is often overlooked.
- More global investment is required to develop and distribute AMR-targeted vaccines, particularly in high-burden regions. It is currently challenging to secure funding for AMR vaccine programmes in low- and middle-income countries.
- Comprehensive data on vaccine impact on AMR are critical and can inform decisions about vaccine financing, introduction and use.

#### International policy perspective – Dr Megan Carey (International AIDS Vaccine Institute)

Megan Carey spoke about the role of international organizations in addressing AMR through vaccination. Key points included the following:

- The potential for vaccines to tackle AMR. Vaccines, like the new tuberculosis (TB) vaccine (MTB VAC), could potentially reduce AMR-associated deaths, disability, hospital costs and antimicrobial use if successful.
- Measuring the effect on AMR requires large sample sizes and diverse AMR strains, making it more suitable for post-introduction studies. Key indicators for AMR impact should include the incidence of drug-resistant infections and antimicrobial use. The MTB VAC trial, for example, will track TB treatment outcomes and antimicrobial use, with the possibility of measuring secondary infections and reduced antimicrobial consumption.
- Understanding baseline AMR distribution in trial sites is crucial for assessing vaccine impact on specific AMR profiles.
- How global partnerships between academia, industry and non-profits can drive vaccine accessibility in high-burden AMR regions.

#### UK policy perspective – Sir Andrew Pollard (Oxford University)

Andrew Pollard provided an overview of the UK’s policy landscape regarding AMR and vaccines. Key points included:

- The UK’s Joint Committee on Vaccination and Immunisation plays a key role in vaccine prioritization; however, AMR is not currently included in its terms of reference, highlighting a gap in policy focus.
- Surveillance and impact assessment. Better data are needed to quantify the reduction in antibiotic use due to vaccines.
- Integrating AMR-focused vaccines into NHS policies, such as ensuring that vaccines that reduce AMR are considered in national immunization schedules.

### Key discussion themes

#### Strengthening the evidence base for vaccines in tackling AMR

- Attendees emphasized the lack of real-world evidence on how effectively vaccines reduce antibiotic use and AMR, with impact assessment hindered by gaps in models and parameters.
- There was discussion on integrating AMR endpoints into vaccination programmes to better measure their impact.
- Current static models underestimate AMR’s evolving impact, making it difficult to assess the true burden and potential impact of vaccines.
- The group highlighted the need for cost-effectiveness models that account for the long-term savings vaccines provide by reducing AMR-related infections.

#### Overcoming policy and regulatory consideration barriers

- Existing vaccine approval frameworks do not explicitly recognize AMR reduction as a benefit, making it difficult for developers to secure funding and regulatory approval for AMR-targeted vaccine.
- The key challenge is not the regulatory agencies themselves but rather aligning regulatory trial requirements with the expectations of funders and policymakers. Recognizing AMR prevention as a legitimate outcome and incorporating it into primary or additional claims would provide clarity and improve alignment across sectors.
- Attendees emphasized the importance of explicitly incorporating vaccines into national and global AMR action plans, with a key takeaway being the need for stronger country-level integration between AMR teams and immunization departments.
- Many low- and middle-income countries face significant barriers to accessing vaccines that could reduce antibiotic use, underscoring the need for international funding and expanded manufacturing initiatives. Strengthening affordability and distribution efforts is essential for improving global vaccine coverage.

#### Encouraging industry engagement and public–private collaboration

- Industry faces significant challenges in embedding the value of AMR into vaccine development. Key concerns include determining which vaccines to prioritize and understanding regulatory expectations.
- To label a vaccine for antibiotic resistance prevention, an efficacy trial would be required, posing a major hurdle. Regulatory uncertainties further complicate efforts to demonstrate the value of AMR-focused vaccines, making it difficult to attract investment and drive development. However, clearly stating the benefits of the potential reduction of AMR on product labels could drive demand and commercial viability if such evidence is generated.
- The discussion highlighted the role of governments and non-profit organizations in de-risking vaccine investment through mechanisms such as advance market commitments.
- Participants stressed that early-stage vaccine research often lacks a clear pathway to market, requiring stronger collaboration between academia, biotech companies and policymakers.

#### Expanding vaccine research priorities

- Whilst bacterial vaccines are a key focus, participants discussed how vaccines for viral infections (e.g. influenza, RSV and COVID-19) could also contribute to AMR reduction by preventing secondary bacterial infections that require antibiotic treatment.
- There was discussion on the need for vaccines in agriculture and veterinary medicine, as antibiotic use in livestock contributes to resistance.
- Targeting high-risk populations (e.g. neonates susceptible to *Klebsiella*) poses a challenge, as these vaccines may be deprioritized due to their smaller target populations.
- Mapping the global genomic diversity of pathogens remains crucial for driving innovation in fundamental vaccinology, enabling the development of more effective and targeted vaccines.

#### The role of the microbiology community in driving change

- Bridging the gap between the AMR and vaccine communities is crucial. Different messaging is required:
  - For the vaccine community: emphasising how vaccines can not only avert death and reduce disease severity, they can also contribute to AMR reduction.
  - For the AMR community: positioning vaccines as a key intervention alongside other AMR reduction strategies.
- Communication gaps within the microbiology community persist, particularly between AMR and TB researchers, limiting collaboration.
- Attendees highlighted the need for microbiologists to engage with policymakers, funders and the public to promote vaccines as AMR solutions. Currently, vaccines are still seen as an alternative approach due to data gaps and communication failures. The WHO’s ‘people-centred approach to addressing AMR in humans [2] was identified as a positive step toward integrating vaccines into global AMR strategies.
- The microbiology community needs to prioritize efforts to encourage vaccine adoption as a key solution to AMR over alternative approaches.
- Encouraging stronger partnerships between microbiologists, epidemiologists, economists and social scientists was seen as essential for making the case for vaccine-mediated AMR reduction.

#### UK strengths

- The UK has strong research and public health infrastructure that can be leveraged for AMR vaccine innovation.
- The UK is a global leader in genomics and vaccine development. Novel approaches could support the development of vaccine candidates that are more cost-effective and efficient.

## Conclusion

The workshop highlighted the transformative role vaccines could play in tackling the AMR crisis, not only by preventing infections but also by reducing antibiotic use and limiting the emergence of resistance. Key themes emerged throughout the day: the urgent need to strengthen the evidence base for vaccine impact on AMR, the importance of enabling regulation and funding for vaccine development and the critical role of interdisciplinary collaboration in translating innovation into policy and practice. Participants were united in calling for vaccines to be elevated from a complementary to a core intervention within AMR strategies.

A particularly strong message was the need to formally recognize the AMR-reducing benefits of vaccines through product labelling. Including AMR impact statements as a proven claim would send a clear signal to funders and healthcare providers about the broader value of vaccines, helping to unlock investment, streamline regulatory approval and accelerate adoption.

The Microbiology Society remains committed to supporting this work. Through the Knocking Out AMR project [3], we will continue to champion vaccines as a cornerstone of sustainable, long-term solutions to AMR, ensuring that the microbiology community leads the way in shaping impactful policy and innovation.

## Abbreviations

AMR: antimicrobial resistance
TB: tuberculosis
WHO: World Health Organization
